# Implementing the 4Ms in a Rural Federally Qualified Health Center: Lessons Learned

**DOI:** 10.1093/geroni/igaa057.2958

**Published:** 2020-12-16

**Authors:** Anna Faul, Joseph D’Ambrosio, Pamela Yankeelov, Sam Cotton

**Affiliations:** 1 University of Louisville Trager Institute, Louisville, Kentucky, United States; 2 University of Louisville, Louisville, Kentucky, United States

## Abstract

The University of Louisville GWEP has partnered with Mountain Comprehensive Care, a FQHC with 9 practices in 5 rural counties in KY, to infuse the 4 M’s of age-friendly health care systems into their daily practices, namely what matters most, medication management, mentation and mobility. Many lessons were learned during this infusion period, specifically related to cultural and rural barriers that make some of these principles very difficult to implement. Specifically, what matters most to older adults in these very poor rural areas may not be the safest way to proceed; medication management may be difficult to do due to the extent of opioid addictions in these areas, mentation has many challenges related to isolation and lack of understanding of dementia and mobility issues are complicated due to the many home barriers to fall safety environments. These lessons will be discussed in this part of the symposium presentation.

